# Supplementary material for: Patterns of Intron Gain and Loss in Fungi
Source: PLoS Biol. 2004 Nov 30;2(12):e422. doi: 10.1371/journal.pbio.0020422 (PMC532390; doi:10.1371/journal.pbio.0020422)
Supplement: Table S1 — Also available at http://genes.mit.edu/NielsenEtAl/. (4.3 MB ZIP). [file pbio.0020422.st001.zip › NielsenEtAl/html/537.html]

AN8401.1.NCU09923.1.MG08985.1.FG07993.1


```
 CLUSTAL W (1.82) Multiple Sequence Alignments - Introns Inserted


Sequence 1: NCU09923.1	790 aa
Sequence 2: FG07993.1	712 aa
Sequence 3: MG08985.1	792 aa
Sequence 4: AN8401.1	763 aa
Alignment Length: 821 aa
Number Identitical Residues: 221 aa
Alignment Score (without introns) 13693


MG08985.1 	--------------MALKYFLAALVGLLP---YIADEVQGAITYPDCENGPLSTNIVCDQ
NCU09923.1	MDGGNRCYSPSITTTNMKSSWASYCLLSCTSALVSAIDLPFQTYPDCVNGPLASLKVCDA
FG07993.1 	-----------MMLHKLQVAASALALLNG----LALAAAPYQQ-PDCTKEPLRSNGICDT
AN8401.1  	--------------MAVFKSWN-LALLSS----LFIPALCQSNYPDCTTGPLSELPICDT
          	                :         *      :          *** . **    :** 

MG08985.1 	AATPAERAAGLVDIMELDEKLENLVN2KSPGAPRIGLPAYEWWSEALHGVAKSPGVTFNK
NCU09923.1	TLSPPQRAAALVAAMTTEEKLQNLVS~KSKGAPRIGLPAYNWWSEALHGVAYAPGTQFRS
FG07993.1 	TASPAERAAALVSALTPREKVNNLVS2NATGAPRIGLPRYNWWNEALHGVAGAPGNDYND
AN8401.1  	SLSPLERAKSLVSALTLEEKINNTGH~EAAGSSRLGLPAYNWWNEALHGVAEKHGVSFEE
          	: :* :** .**  :   **::*    :: *:.*:*** *:**.*******   *  :..

MG08985.1 	SSGAAFSSATSFSNPIVLSAAFDDELVEAVATQISTEARAFSNAGLAG-LDWWTPNINPY
NCU09923.1	G-DGPFNSSTSFPMPLLMAATFDDELIEKVGEVIGTEGRAFGNAGFSG-FDYWTPNVNPF
FG07993.1 	--KPPYDSATSFPMPLLMGSTFDDDLIHDIGEVIGTEARAWNNGGWGGGVDYWTPNVNPF
AN8401.1  	S--GDFSYATSFPAPIVLGAAFNDALIRRVAEIISTEARAFSNSDHAG-IDYWTPNVNPF
          	.    :. :***. *:::.::*:* *:. :.  *.**.**:.*.. .* .*:****:**:

MG08985.1 	KDPRWGRGMETPGEDALRISKYVKAL~LRGLEGSDPTTRKMVANCKHYAANDLERWNGVT
NCU09923.1	KDPRWGRGSETPGEDILRIKRYAASM~IRGLQGP-LPERRVVATCKHYAANDFEDWNGST
FG07993.1 	KDPRWGRGSETPGEDALHVSRYARAM0E--------------------------------
AN8401.1  	KDPRWGRGQETPGEDPLHCSRYVKEF~VGGLQGDDPEKPKVVATCKHLAAYDLEEWGGVS
          	******** ****** *: .:*.  :   . .. .       :...  :: . .  .. :

MG08985.1 	RYNFDAPVTLQDLSEYYLPAFKQCARDSNVGSFMCAYNAMSIKGKDLSWNGTPVCASKYL
NCU09923.1	RHDFDAKVTLQDLAEYYLSPFQQCARDSKVGSIMCSYNAV---------NGVPACANTYL
FG07993.1 	-----------------------CTRDAKVGSIMCSYNAV---------NGIPACANSYL
AN8401.1  	RFEFDAKVSAVDLLEYYLPPFKTCAVDASVGAFMCSYNAL---------NGVPACADRYL
          	  . .:  :  .  .   .. . *: *:.**::**:***:         ** *.**. **

MG08985.1 	MNDILREHWGWKEHNNWITSDCNAVLH1MWNQHHWSDTREEAAGSAYTAGTDTVCEVSNY
NCU09923.1	MQTILREHWNWTAPGNYITSDCEAVLD~IFANHHYAKTNAEGTALAFEAGTDSSCEYE--
FG07993.1 	QETLLRKHWNWTHTNNWITSDCGAMQD~IWQHHNYTKTGAEAAKAAFENGQDSSCEYT--
AN8401.1  	LQTVLREHWGWEGPGHWVTGDCGAVER~IQTYHHYVESGPEAAAAALNAGVDLDCGTW--
          	 : :**:**.*   .:::*.** *:   :   *:: .:  *.:  *   * *  *     

MG08985.1 	DKTAVKGAFDRGLLDEDVVDRALKRLYEGLVRAGYFD-GPDAPYRNITWADVNTPEARKL
NCU09923.1	SSSDIPGAWTQGLLEQSTVDRALTRLYEGLVRVGYFD-GNHSEYASLGWKDVNSPKSQEV
FG07993.1 	TTKDISDSYEQGLLTEKVMDRALKRLFEGLVHTGFFD-GDKSEWSSLDFDDVNTRHAQDL
AN8401.1  	LPSYLGEAERQGLISNETLDAALTRLYTSLVQLGYFDPAEGQPLRSLGWDDVATSEAEEL
          	  . :  :  :**: :..:* **.**: .**: *:**..      .: : ** : .:..:

MG08985.1 	AHRSAVEGMVLTKN-NGVLPIKLEELQKKGKTVALIGNWVDNGEQMLGTYSGIAPFRNTP
NCU09923.1	ALQTAVEGIVLLKN-DQTLPLGLKTDP--KSKLAMIGFWANDPKTLSGGYSGKPAFEHSP
FG07993.1 	ALQSAVRGAVLLKN-DNTLPLNIKK----KESVALIGFWADDKTKLQGGYSGPAPHVRTP
AN8401.1  	AKTVAIQGTVLLKNIDWTLPLKANG------TLALIGPFINFTTELQSNYAGPAKHIPTM
          	*   *:.* ** ** : .**:  :       .:*:** : :    : . *:* . .  : 

MG08985.1 	LAAAKALNLKMVTAGGPVNQSTGSRDSWTRPALNAAIQADVVLYFGGIDLSVEAEDRDRY
NCU09923.1	VYAAEAMGFNVTTAGGPVLQNSTSNDTWTQAALEAAQDANYILYFGGLDTSAAGETKDRT
FG07993.1 	AYAAKMLGLNTNVAWGPTLQNSSVPDNWTTNALEAAKKSDYIVYLGGLDATAAGEERDRT
AN8401.1  	IEAAERLGYNVLTAPGTEVNSTSTDG--FDDALAIAAEADALIFFGGIDNTVEEESLDRT
          	  **: :. :  .* *.  :.:   .     **  * .:: ::::**:* :.  *  ** 

MG08985.1 	SLAWPSAQAKLLSDISALGKPTVVVQLG-TMLDDTALLDNKNISAIIWAGYPGQDGGTAA
NCU09923.1	TINWPEAQLQLIKTLTKLGKPLVVVQMG-DQLDNTPLLATKTVNSILWANWPGQDGGTAV
FG07993.1 	DLDWPSTQLTLLKKLSNLGKPLVVVQLG-DQVDDTPLLKNKGVNSILWVNYPGQEGGTAV
AN8401.1  	RIDWPGNQEELILELAELGRPLTVVQFGGGQVDDSALLASAGVGAIVWAGYPSQAGGAGV
          	 : **  *  *:  :: **:* .***:*.  :*::.** .  :.:*:*..:*.* **:..

MG08985.1 	FDIITGKTAPSGRLPVTQYPAKYANQVPMTDMEVRPSKDTKGGAASNPGRTYRWYDEAVH
NCU09923.1	MQILTGLKSPAGRLPVTQYPANYTAAVPMTDMNLRPSD-------RLPGRTYRWYPTAVQ
FG07993.1 	MELITGRKGPAGRLPLTQYPSKYTEQVGMLEMELRPTK-------SSPGRTYRWYSDSVL
AN8401.1  	FDVLTGKAAPAGRLPITQYPKSYVDEVPMTDMNLQPGT-------DNPGRTYRWYEDAVL
          	::::**  .*:****:**** .*.  * * :*:::*           ********  :* 

MG08985.1 	PFGFGLHFTNFTTSVAVSSSSAISTSDLESGCKSEK---HIDKCSFPSSLEVSVTNDGKS
NCU09923.1	PFGFGLHYTTFQAKIAAPL-PRLAIQDLLSRCGGDNANAYPDTCALPP-LKVEVTNSGN-
FG07993.1 	PFGFGKHYTTFKAMFKSQK-IEMNIQKILKGCDAT----YVDTCPLPP-IHLSVKNTGR-
AN8401.1  	PFGFGLHYTTFNVSWAKKAFGPYDAATLARGKNPSS--------NIVDTFSLAVTNTGD-
          	***** *:*.* .              :       .         :  :: : *.* *  

MG08985.1 	TTSSYAALAFVRG-EYGPKPYPLKTLVAYGKLHDIAPGQTKKVKLELTLGDLARTAENGD
NCU09923.1	RSSDYVVLAFLAG-DAGPRPYPIKTLVSYTRLRDVSPGHKTTAHLEWTLGDIARYDEQGN
FG07993.1 	TTSDFVSLVFIQG-KVGPKPYPLKTLAAYSRSHDIKPRATKDVELQWTMDNIARREKNGD
AN8401.1  	VASDYVALVFASAPELGAQPAPIKTLVGYSRASLIKPGETRKVDVEVTVAPLTRATEDGR
          	 :*.:. *.*  ... *.:* *:***..* :   : *  .  ..:: *:  ::*  ::* 

MG08985.1 	LVLYPGKYEVLVDVP-TAAKASFEIRGGELVLDRFPQPKDD---
NCU09923.1	TVLYPGTYTVTVDEP-AQASASFVVEGEAVVLDRWPAPSGQVVV
FG07993.1 	LVVYPGTYTLLLDEP-TQVKIKVKLTGDKVVLDRWPEFHGSD--
AN8401.1  	VVLYPGEYTLLVDVNDEYPTAKFEIKGDVQVLEKFPLSGNDSD-
          	 *:*** * : :*  .   . .. : *   **:::*   ..
```
